# The Evolution of Endophagy in Herbivorous Insects

**DOI:** 10.3389/fpls.2020.581816

**Published:** 2020-11-02

**Authors:** John F. Tooker, David Giron

**Affiliations:** ^1^Department of Entomology, The Pennsylvania State University, University Park, PA, United States; ^2^Institut de Recherche sur la Biologie de l’Insecte, UMR 7261, CNRS/Université de Tours, Parc Grandmont, Tours, France

**Keywords:** Coleoptera, Diptera, gall-inducing insect, Hemiptera, Hymenoptera, leaf-mining insect, Lepidoptera, Thysanoptera

## Abstract

Herbivorous feeding inside plant tissues, or endophagy, is a common lifestyle across Insecta, and occurs in insect taxa that bore, roll, tie, mine, gall, or otherwise modify plant tissues so that the tissues surround the insects while they are feeding. Some researchers have developed hypotheses to explain the adaptive significance of certain endophytic lifestyles (e.g., miners or gallers), but we are unaware of previous efforts to broadly characterize the adaptive significance of endophagy more generally. To fill this knowledge gap, we characterized the limited set of evolutionary selection pressures that could have encouraged phytophagous insects to feed inside plants, and then consider how these factors align with evidence for endophagy in the evolutionary history of orders of herbivorous insects. Reviewing the occurrence of endophytic taxa of various feeding guilds reveals that the pattern of evolution of endophagy varies strongly among insect orders, in some cases being an ancestral trait (e.g., Coleoptera and Lepidoptera) while being more derived in others (e.g., Diptera). Despite the large diversity of endophagous lifestyles and evolutionary trajectories that have led to endophagy in insects, our consideration of selection pressures leads us to hypothesize that nutritionally based factors may have had a stronger influence on evolution of endophagy than other factors, but that competition, water conservation, and natural enemies may have played significant roles in the development of endophagy.

## Introduction

Among insects, feeding within plant tissue, or endophagy, has evolved numerous times and is one of the major feeding strategies for herbivorous insects. Guilds of endophytic feeders include borers, miners, and gall inducers and inquilines, but allied taxa, such as leaf tiers and leaf rollers, also tend to be included in the guild because they all have concealed feeding habits associated with plants. Endophytic associations of insects and their host plants can be millions of years old and are evident in the fossil record. For example, wood boring, leaf mining and insect galls have all been recorded from Carboniferous deposits and may have even evolved earlier (∼300 million years ago; Chaloner et al., 1991; Labandeira and Phillips, 1996; Feng et al., 2017). Additionally, there are even some extant endophytic taxa evident in fossils, with good examples provided by the lepidopterans *Ectodemia* and *Stigmella*, and the aphid *Melaphis rhois*, suggesting that the interactions of these taxa with their host plants are 97- and 48-million years old, respectively (Moran, 1989; Labandeira et al., 1994).

In some taxa, feeding within plant tissue appears to have been an ancestral state, whereas in others endophagy appears to be derived. In still others, specialized endophagy has developed even further, into an extremely sophisticated form of feeding, occasionally involving mutualistic symbionts. For example, galls and mines represent extended phenotypes of the insect species that induce or form them; these structures result from complex interactions among genomes of the host plant, insect, and, sometimes, their symbionts (Giron et al., 2016).

Despite the ubiquity of endophagy within Insecta, we are unaware of any previous effort in the ecological or systematics literature to broadly delimit the guild across taxa and characterize the limited set of selective forces that could have facilitated evolution and diversification of endophytic feeding habits. Certainly some publications have address a single taxon (e.g., flies; Labandeira, 2005) or characterized the adaptive significance of a particular form of endophagy (e.g., leaf mining and gall inducing; Price et al., 1987; Connor and Taverner, 1997; Stone and Schönrogge, 2003), but these publications did not generally consider endophagy beyond these specific guilds, nor did they consider the evolution of endophagy in a comparative framework.

From just reading classical literature that often forms the basis of ecological courses, one could easily get the impression that most taxa have followed a simple progression as typified by some model systems, from exposed leaf feeding to endophagy to more specialized forms of endophagy like mining and/or galling (Price et al., 1987; Price, 1992; Nyman et al., 1998, 2000). However, the systematics literature, which is often not closely tracked by ecologists, reveals that evolution of endophagy varies greatly by taxa, so a diversity of selection pressures must have been involved in its evolution and diversification. Highlighting the presumed evolutionary sequence leading to endophagy in each insect order can provide insights on selection pressures that could have played a role on its evolution. And comparing evolutionary pathways in these various endophagous feeding guilds can provide evidence about which of these selective forces may have played major evolutionary roles, allowing us to formulate hypotheses that could be tested with quantitative methods.

Our goal in this paper is to consider via existing literature the selection pressures that could have played a role in evolution and diversification of endophagy within Insecta. We will begin by defining endophagy and generally describing its occurrence across Insecta. We then will discuss the selection pressures that could have encouraged various groups of insects to develop a concealed feeding habit and subsequently diversify. The six selection pressures we selected are drawn from previous literature with herbivorous insects generally (Strong et al., 1984) and gall insects and leaf miners, more specifically (Price et al., 1987; Connor and Taverner, 1997). They have support from particular taxa, but have not been discussed previously in terms of general endophagy. By considering endophagy generally, we aim to stimulate hypotheses that can be tested in specific groups or through comparative studies that seek to clarify selection pressures associated with the adaptive significance of various forms of endophagy. In considering evolutionary selection pressures that could have facilitated endophagy, we do not address neutral evolutionary processes (e.g., genetic drift), which can influence patterns of evolution in some insect taxa but would require a quantitative analysis of taxa and their feeding styles (Peterson et al., 2016), which is beyond the scope this paper. We also do not aim to comment on the latest developments from a molecular perspective concerning the evolution of herbivory and feeding specialization (Groen and Whiteman, 2016). Rather, we use published phylogenies to illustrate the diversity of patterns of endophagy among insect orders and how they can diverge from the simplistic views held by ecologists. After considering the possible selection pressures, we will broadly characterize occurrence of endophagy across orders of herbivorous insects and discuss which selection pressures could have been active for these taxa. We finish by considering which selection pressures may have been most relevant for evolution of the endophytic habitat generally.

## Endophagy

The definition of “endophagy” or “endophytic feeding” that we will use in this paper is “insect feeding on plant or fungal tissues that occurs within tissue of a living plant, whether the specific plant tissue is live or dead.” This definition allows us to include insects that feed upon non-living portions of living plants, such as bark, heartwood, and pith, but excludes insects that mostly feed upon dead or decaying plants (i.e., decomposers or detritivores, like termites; Weesner, 1960) or those that live inside plants but do not eat them (e.g., Edwards et al., 2009). Consistent with previous assessments (Labandeira, 2005), we also include seed feeders (i.e., seed predators) whose endophytic larvae consume seeds prior to seed dispersal (Janzen, 1971). The definition is not perfect because some taxa, particularly in Coleoptera (e.g., Cerambycidae), contain species that feed in live plants while others that feed in dead plants. In cases like this where the taxon falls into some gray area near our definition, we try to include them to provide appropriate context and acknowledge that biological continuums can be difficult to divide into perfect bins. Lastly, to be considered an endophagous feeder, a taxon needs just one life stage to feed endophagously. Most commonly, larvae or nymphs are the concealed feeders but adults of some taxa are also endophytic (e.g., bark beetles [Curculionidae: Scolytinae], or aphids [Aphididae] or thrips [Thysanoptera] that develop in galls). We are not aware of any taxa in which immature stages are not endophytic but adults are.

We avoid the term “endophytophagy” because others have accepted the term “phytophagy” to mean “feeding on living tissue of higher plants” (“higher plants” being a synonym for “vascular plants”; Strong et al., 1984; Mitter et al., 1988) and we want to include in our discussion insects that have found a way to live in and feed upon any plant or fungal tissue of a live plant. By focusing on plant or fungal feeding, our definition includes mutualisms between insects and fungi (e.g., ambrosia galls) in which the insect indirectly feeds upon plants by eating fungi, which consume the plant; these often-symbiotic fungi have facilitated endophytic lifestyles for some taxa (e.g., Hymenoptera, Coleoptera, Diptera; Bissett and Borkent, 1988; Hanson, 1995; Farrell, 1998; Heath and Stireman, 2010). Our definition excludes predation or parasitoidism, animal-animal interactions which can occur within plant tissue but are obviously not plant feeding.

This definition will permit us to consider a full range of herbivorous insects with concealed lifestyles, including borers, miners, gall inducers, inquilines, and leaf rollers, tiers, and webbers. Borers and miners are similar and appear to be informally distinguished from one another based on their depth away from plant surfaces or tissue layers, with miners being close to the surface and borers being deeper. More formally, mines have been defined as “feeding channels caused by insect larvae inside the parenchyma or epidermis of plants, in which its outer wall remains undamaged, thus shutting off the mine activity from outside” (Hering, 1951). Mining can occur in bark, cambium, flowers, fruits, leaves, and stems (Powell et al., 1998), and comes in different shapes and sizes (e.g., linear, digitate, blotch or tentiform mines, among others) that tend to be species specific (Eiseman, 2020). Borers (sometimes known as tunnelers) can feed upon tissues of live trees, such as cambium, pith or wood in trunks, branches, shoots, stems, and roots, but borers can also attack flowers, fruits, and seeds (Solomon, 1995; Powell et al., 1998). Broadly speaking, gall inducers can also attack a range of plant tissues, but as a group they typically oviposit into, or feed upon, meristematically active tissues to force production of their galls (Raman et al., 2005). Some gall insects can even induce meristematically active tissues (Ananthakrishnan, 1992), which is an impressive accomplishment without an obvious mechanism. At the species level, many endophytic insects, particularly gallers and leaf miners, are monophagous and attack specific plant tissues at a specific plant-developmental stages (Connor and Taverner, 1997; Raman et al., 2005; Giron et al., 2016). Though they may appear outwardly similar, leaf-roller species take one of two approaches to hide: those that use silk to roll the leaf and others that induce tissue proliferation (“roll galls”) by feeding upon one side of the leaf, leading to rolling (Dreger-Jauffret and Shorthouse, 1992).

**FIGURE 1:**
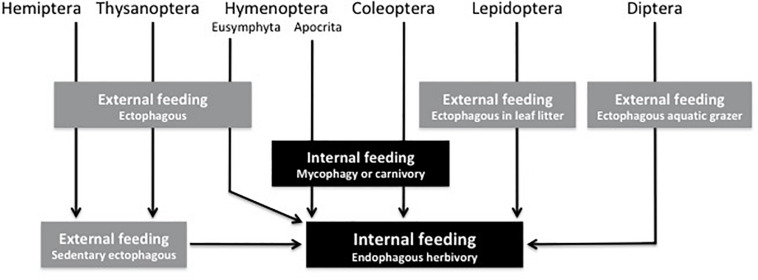
The ancestral feeding modes of six herbivorous insect orders and how they led to endophagy.

Endophytic insects are concentrated in six of the largest orders of Insecta: Thysanoptera, Hemiptera, Hymenoptera, Coleoptera, Lepidoptera, and Diptera (Table 1). Of all phytophagous orders, Orthoptera and Phasmatodea do not appear to have any endophagous taxa. We will briefly address in phylogenetic order the occurrence of endophagy in these large orders (Grimaldi and Engel, 2005; Peters et al., 2014), and later we will return to these taxa to consider specific selection pressures that likely influenced evolution of endophagy in these groups of animals. The routes to endophagy for some orders are similar, but others took different paths (Figure 1).

**TABLE 1:** Taxa of plant-feeding insects that include significant endophagous species.

Order	Percent herbivorous spp.	Types of endophytic feeders	Notable taxa with significant endophytic species
Thysanoptera	68	Gall inducers	Phlaeothripidae^G^
		Gall inquilines	
Hemiptera	78	Gall inducers	Aphidoidea
		Gall inquilines	Aphididae^G^
			Coccoidea
			Asterolecaniidae^G^
			Beesoniidae^G^
			Eriococcidae^G^
			Phylloxeroidea
			Phyllorxeridae^G^
			Adelgidae^G^
			Psylloidea
			Calophyidae^G^
			Phacopteronidae^G^
			Psyllidae^G^
			Tingoidea
			Tingidae^G^
Hymenoptera	7	Borers	**Symphyta**
		Leaf folders	Pamphilioidea
		Leaf rollers	Pamphiliidae
		Leaf miners	Tenthredinoidea
		Gall inducers	Tenthridinidae^G^
		Gall inquilines	Siricoidea
			Siricidae
			Cephoidea
			Cephidae
			**Apocrita**
			Ichneumonoidea
			Braconidae^G^
			Chalcidoidea
			Agaonidae^G^
			Eulophidae^G^
			Eurytomidae^G^
			Pteromalidae^G^
			Tanaostigmatidae^G^
			Torymidae^G^
			Cynipoidea
			Cynipidae^G^
Coleoptera	26	Borers	Buprestoidea
		Miners	Buprestidae^L,G^
		Gall inducers	Elateroidea
		Gall inquilines	Elateridae
			Lycidae
			Bostrichoidea
			Bostrichidae
			Anobiidae
			Tenebrionoidea
			Mordellidae
			Tenebrionidae
			Chrysomeloidea
			Cerambyciidae^G^
			Chrysomelidae^G,L^
			Curculionoidea
			Anthribidae
			Attelabidae^L^
			Brentidae^G^
			Curculionidae^G^
Lepidoptera	100	Borers	Nepticuloidea
		Leaf folders	Nepticulidae^G,L^
		Leaf rollers	Gracillarioidea
		Leaf tiers	Gracillariidae^G,L^
		Leaf miners	Yponomeutoidea
		Gall inducers	Glyphipterigidae^G,L^
		Gall inquilines	Gelechioidea
		Leaf mine-gallers	Cosmopterigidae^G,L^
		Leaf Mine-rollers	Depressariidae
			Elachistidae^G,L^
			Gelechiidae^G,L^
			Sesioidea
			Sesiidae^G^
			Cossoidea
			Cossidae
			Tortricoidea
			Tortricidae^G^
			Pterophoroidea
			Pterophoridae^G,L^
			Pyraloidea
			Crambidae^G^
			Pyralidae
			Thyridoidea
			Thyrididae^G^
			Noctuoidea
			Noctuidae
Diptera	28	Borers	**Nematocera**
		Leaf miners	Sciaroidea
		Gall inducers	Sciaridae^L^
		Gall inquilines	Cecidomyiidae^G, L^
			Chironomoidea
			Chironomidae^L^
			Ceratopogonidae^L^
			**Brachycera**
			Stratiomyoidea
			Pantophthalmidae
			Xylomyidae
			Asiloidea
			Asilidae
			Empidoidea
			Dolichopodidae^L^
			Platypezoidea
			Phoridae^L^
			Syrphoidea
			Syrphidae^L^
			**Schizophora-Acalyptratae**
			Diopsoidea
			Psilidae^L^
			Tephritoidea
			Lonchaeidae^G^
			Tephritidae^G,L^
			Opomyzoiea
			Agromyzidae^G,L^
			Fergusoninidae^G^
			Lauxanioidea
			Lauxaniidae^G,L^
			Ephydroidea
			Drosophilidae^L^
			Ephydridae^L^
			Chloropidae^G,L^
			**Schizophora-Calyptratae**
			Muscoidea
			Anthomyiidae^G,L^
			Scathophagidae^L^

*In most taxa, nymphs or larvae are the endophytic life stage. Orders with fewer endophytic taxa have most of their endophytic families listed, whereas space limitations prevents listing of all endophytic families. Endophytic designations based on various edited volumes (McAlpine et al., 1981, 1987; Solomon, 1995; Powell et al., 1998; Arnett and Thomas, 2000; Arnett et al., 2002; Labandeira, 2005; Raman et al., 2005). Groups marked with a superscript G or L include gall-inducing or leaf-mining species. Percent herbivorous species is taken from Wiens et al. (2015).*

The hemipteroid orders Thysanoptera and Hemiptera have evolved limited forms of endophagy. For both groups, plant-fluid feeding was a key innovation that appears to contributed to their success (Johnson et al., 2018), but this mode of feeding must have limited their ability to evolve different modes of endophagy—sucking mouthparts facilitate injection of effectors stimulating the proliferation of new plant tissues around the insect but may also restrict their ability to enter plant tissue. As a result, the only recorded endophytic species in these orders feed between attached leaves, induce galls, or are inquilines that exploit these feeding sites (Table 2; Ananthakrishnan, 1992; Burckhardt, 2005; Gullan et al., 2005; Mound and Morris, 2005). Members of these two orders, of course, have incomplete metamorphosis; therefore, immature stages have the same form of feeding as adults. Compared to holometabolous taxa, in which larvae and adults have often evolved different forms of feeding, hemimetabolous metamorphosis may have in part constrained the forms of endophytic feeding that could have evolved in these two orders.

**TABLE 2:**
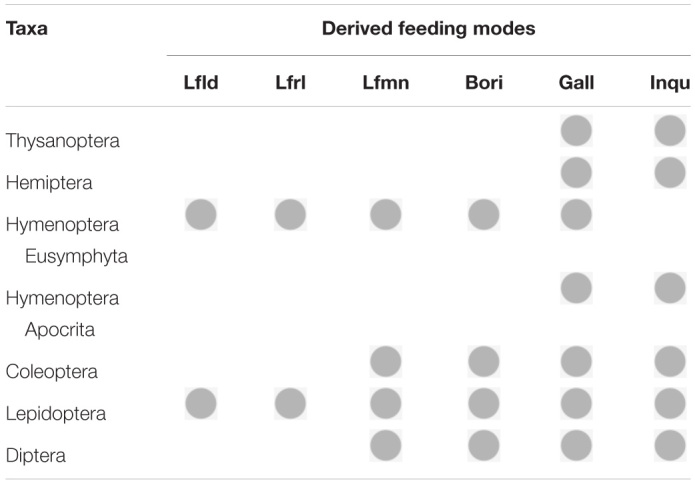
Endophagous feeding modes that evolved in each herbivorous order.

*Abbreviations for derived feeding mode are: Lfld, leaf folding; Lfrl, leaf rolling; Lfmn, leaf mining; Bori, boring; Gall, galling; Inqu, inquilines.*

In contrast to hemimetabolous groups, holometabolous groups have benefited from the diets and feeding styles that can evolve differently in larvae and adults. Indeed, endophagy among holometabolous groups occurs mainly in larval stages, and evolution of complete metamorphosis may be tied to concealed feeding niches (Grimaldi and Engel, 2005). Current evidence suggests that holometabolous insects may have evolved from an ancestor with an orthognathous head and chewing mouthparts that fed externally on plants or fungi (Peters et al., 2014). Moreover, the common ancestor of Coleoptera, Lepidoptera, and Diptera (among other taxa in Aparaglossata) may have had prognathous heads, which would have facilitated burrowing into substrates (Peters et al., 2014). Chewing mouthparts on orthognathus or prognathous heads may have been key innovations in the evolution of endophagy in Hymenoptera, Coleoptera, Lepidoptera and Diptera, allowing them to chew into plant tissue and evolve a diversity of endophytic feeding modes (Labandeira, 1997). Generally, it seems that evolution of mouthparts is key in evolution of feeding habits, allowing transitions from ecto- to endophagy but also the diversification of endophagous feeding modes (Body et al., 2015; Guiguet et al., 2019).

Endophagy of Hymenoptera and Coleoptera may have been initially facilitated by fungi (Sharkey, 2007; Massini et al., 2012), but endophytic feeding in both groups extends well beyond fungus feeding and they contain wide varieties of endophytic feeders (Table 2). The evolutionary trajectory of two major clades of Hymenoptera, Eusymphyta and Apocrita, has resulted in different diversities of endophytic species in the two groups (Table 2). Coleoptera has a limited range of endophytic taxa compared to Hymenoptera and Lepidoptera, but the abundance of endophagy in Coleoptera is remarkable; larvae of particularly speciose taxa, like Curculionidae and Cerambycidae, are almost exclusively endophytic (Turnbow and Thomas, 2002; Oberprieler et al., 2007). Lepidoptera appears to host the most diverse array of endophytic habits, in part because endophagy developed early in the evolution of the group (Powell et al., 1998). Diptera has evolved a diversity of endophytic habits, and endophagy is particularly important for flies because it is practically the only form of herbivory within the group (Labandeira, 2005).

## Selection Pressures Leading to Evolution of Endophytic Feeding

Most evidence suggests that endophagy has evolved repeatedly in most of the dominant orders of herbivorous insects (see below for details). In some orders (Hemiptera, Thysanoptera), diversification of endophagy has been limited to few modes of feeding and relatively few families contain endophagous members, while in others (Lepidoptera, Coleoptera, Diptera, and Hymenoptera) modes of endophagy are more diverse and there appear to be more abundant taxa that have evolved endophagy (Table 1). Obviously, endophagy can be a successful method of feeding on plants even if in some respects it can constrain diversification (Powell et al., 1998). The question we want to address is “why did it evolve so frequently?” In other words, what are the advantages of endophagy, and what active selection pressures could have facilitated its evolution?

Over evolutionary time, insect herbivores have had to overcome several challenges to use plants as food sources. Four primary challenges that have been proposed are attachment (i.e., remaining on plants), desiccation, nourishment, and plant defenses (Strong et al., 1984), all of which could have been selection pressures that encouraged the evolution of endophagy. To this list of challenges that needed to be overcome for herbivores to be successful, we add two more, natural enemies and competition (Price et al., 1987; Denno et al., 1995); therefore, we will consider a total of six challenges that may have played a role in encouraging insects to feed within plant tissue. Three of these factors (desiccation, nutrition, and natural enemies) have previously been identified as selection pressures that likely contributed to the evolution of galling and leaf mining; these three factors have been formulated into hypotheses known as the microenvironment, nutrition, and natural enemy hypotheses (or something similar; Price et al., 1987; Connor and Taverner, 1997; Stone and Schönrogge, 2003). In addition to being involved in evolution of gall induction and leaf miners, these three selection pressures are also relevant to the broader topic of the evolution of endophagy. We will relate each of these six factors to endophagy, and consider how endophytic feeding may have mitigated some of the challenges of herbivory. Of these factors, we first address attachment, desiccation and natural enemies, all of which deal with mortality external to plants. Next, we address nourishment and plant defenses together because these two intertwined issues relate to food intake. Lastly, we address competition, which appears to have been largely overlooked as a potential factor that could have facilitated endophagy or its diversification.

### Attachment

Staying attached to their host plants is a challenge faced by external-feeding herbivores. Plants surfaces can be hairy, spiny, or waxy, making it difficult for herbivores to keep hold of plants. Insects, however, have evolved various adaptations for grasping plants, including abdominal prolegs, crochets, empodia, and various setae (Strong et al., 1984). In contrast, many endophytic insects, particularly borers and gall inducers, face minimal challenges of attachment because the parts of the plants that they attack (e.g., roots, stems, and branches) are usually well integrated into the plant. Moreover, even eggs and immature insects of many endophytic taxa face little risk of falling off the plant because their mothers insert eggs into plant tissue, then upon hatching the insects begin feeding endophytically, with little or no exposure to the external environment (e.g., Whiteman et al., 2011). We do not mean to imply that boring into plants is easier than holding on to the outside of them, but key morphological adaptations and specific traits (e.g., chewing mouthparts, plant-penetrating ovipositor) could have facilitated evolution and diversification of endophagy (Body et al., 2015; Pelaez et al., 2020), decreasing the challenge of attachment.

As evidence that selection pressures can encourage some insects to remain attached to plants, consider leaf miners and leaf gallers. These guilds of insects face the risk of abscission should their host shed leaves prematurely (Williams and Whitham, 1986; Stiling and Simberloff, 1989; Connor and Taverner, 1997). To counteract this risk, some leaf miners have evolved an ability to prevent leaf abscission by modulating phytohormone levels (Zhang et al., 2016), while others can maintain the photosynthetic activity of their host leaves, which could mitigate some effects of premature leaf drop (Giron et al., 2007; Kaiser et al., 2010).

### Desiccation/Microenvironment

Desiccation is a general concern for insects, but it is particularly relevant for species that feed externally on plants because they are exposed to wind and solar radiation, which can dry them out quickly. To combat desiccation, insect species have evolved methods to counteract or minimize water loss, including actively drinking water or positioning themselves on parts of plants with the highest humidity (Strong et al., 1984). Other insect taxa have evolved tactics that modify their immediate surroundings by folding or rolling leaves, living within plant tissue (i.e., mining or boring) or creating new tissues to live in (i.e., galling; Strong et al., 1984), but the role of desiccation prevention in evolution of these endophagous traits is not clear. Nevertheless, endophytic insects and their eggs, which are often embedded in plant tissues, are likely to benefit from being encased in water-filled plant tissue that likely protects them from the drying effects of sun and wind. Being surrounded by water-filled tissue would be particularly important for small, immature stages, which are most vulnerable to water loss (Strong et al., 1984). Moreover, endophagous larvae can benefit physiologically from associating with water-rich tissues, which can simultaneously increase O_2_ and decrease CO_2_ concentrations near larvae, preventing risks of hypoxia or hypercarbia (Pincebourde and Casas, 2016). As mentioned elsewhere in this paper, herbivory in Diptera has evolved almost exclusively in moist, endophytic situations (Dempewolf, 2005), with its taxa likely thriving due to their intimate association with moist tissues or habitats.

The importance of internal feeding for tolerance of desiccation is supported by patterns of galling that show that there are more galls in hotter and drier parts of the world (Price et al., 1987; Fernandes and Price, 1988; Ananthakrishnan, 1992). Gall diversity is also found to be higher in hotter and/or drier environments, like deserts or the upper canopy of Amazonian forests, where leaf temperatures can reach lethal limits (Price et al., 1998; Julião et al., 2014). Similar surveys seem to be lacking for most other endophytic taxa. For leaf miners, some studies have found no association between abundance of leaf-miner species and rainfall, whereas others have found more leaf miners in xeric sites (Sinclair and Hughes, 2010). Experimental evidence, however, indicates that temperatures inside mines are up to 8°C cooler than those on the exposed leaf surface, and can differ from atmospheric temperature by up to 13°C (Pincebourde and Casas, 2006; Pincebourde et al., 2007). Such data suggest that insects in mines would experience lower temperatures, which should relate to lower rates of water loss, but other advantages related to mines preventing desiccation have not emerged (Connor and Taverner, 1997).

### Natural Enemies

Feeding inside plant tissues appears to provide some protection from natural enemies simply because, compared to ectophytic species, endophytic insects appear harder to find and access. From an evolutionary perspective, the first insects that found their ways inside plant tissues likely had selective advantages within populations if they suffered less mortality from predators, parasitoids, and pathogens, possibly facilitating evolution of endophagy. Natural enemies have previously been hypothesized as factors that may have selected for endophytic lifestyles (e.g., leaf mining and galling; Price et al., 1987; Connor and Taverner, 1997; Stone and Schönrogge, 2003). While support for these hypotheses has not been uniform across taxa, endophytic life styles generally appear to be less susceptible to natural-enemy-induced mortality (Cornell and Hawkins, 1995). Analyses of life tables have revealed that some endophytic life stages or groups of insects tend to be attacked less by natural enemies than external feeding species (Cornell and Hawkins, 1995). In particular, eggs of endophytic insects, which tend to be inserted into plant tissues, are killed significantly less often by predators than eggs of ectophytic insect taxa, which tend to be deposited on plant surfaces (Hawkins et al., 1997). (The lower egg mortality rates of endophytic insects may also arise because internal-feeding species tend to lay small and inconspicuous eggs while external feeders often lay eggs in clusters; Connor and Taverner, 1997). Similarly, borers, root feeders, and gallers generally appear to suffer significantly less mortality from predators and pathogens than exophytic species, while also gaining some protection from parasitoids by being hidden inside tissue (Hawkins et al., 1997; see below for exceptions associated with parasitoids). Moreover, at least one group of gall inducers shows strong support for the benefit of endophagy for protection against natural enemies. The mean number of parasitoids attacking nematine sawflies decreased steadily from those that attack external feeders to leaf gallers and finally to shoot gallers, suggesting that more concealed insects suffer less mortality (Price and Pschorn-Walcher, 1988). Compared to external feeders, leaf miners also appear to gain some protection from feeding within plant tissue because they appear to suffer very little mortality from pathogens and significantly less mortality from predators, likely because miners are not usually exposed to the external environment (Connor and Taverner, 1997; Hawkins et al., 1997).

Generally concealed feeders gain protection from natural enemies, but notable exceptions emerge when considering mortality from hymenopteran parasitoids, which tend to have specialized ovipositors that can reach hosts hidden in plant tissues. Compared to leaf rollers, borers, and root feeders, leaf-mining larvae suffer significantly higher mortality from parasitoids (Connor and Taverner, 1997; Hawkins et al., 1997). Moreover, classical biological control programs have been successful against exotic leaf-mining species, indicating parasitoids can severely limit leaf-miner success (Sinclair and Hughes, 2010). Similar to leaf miners, some gall-insect taxa tend to suffer similar mortality from parasitoids as exposed-feeding taxa (Hawkins et al., 1997; Stone and Schönrogge, 2003). This higher mortality of miners and gallers may be driven in part by visual cues associated with most leaf mines and galls, which tend to be obvious (at least to some visual systems), perhaps facilitating their location by parasitoids. Moreover, parasitoids can generally learn to associate rewards with shapes (Wäckers and Lewis, 1999) and some parasitoid species preferentially land on mined leaves (Godfray, 1994). Parasitoids, of course, can also use vibratory and chemical cues to find their hosts. In some endophytic systems, these types of cues can attract parasitoid wasps or help parasitoids localize the host in its hidden microhabitat (Djemai et al., 2001, 2004; Tooker and Hanks, 2006), but in other systems such cues may not be available (Tooker and De Moraes, 2007; Tooker et al., 2008; Hall et al., 2017). Therefore, it may be that cues associated with other endophytic guilds are more challenging for parasitoids to exploit than cues from mines and galls.

### Nourishment and Plant Defenses

Endophagous organisms are peculiar for several reasons. First, most display high levels of fidelity to specific organs of particular host-plant species, although a few appear to have some flexibility across related plant species (Hering, 1951; Raman et al., 2005). This evolved selectivity may have allowed insects to consume the optimal food from among the available plant species in their environment. Feeding inside plant tissues also appears to provide some nutritional advantages simply because endophytic insects can avoid highly defended, outer layers of plant tissue and access nutritionally rich inner plant tissues. Many endophagous insects only consume certain tissues or cell types and reject others. This provides them with the unique scenario of consuming high-quality tissues in an otherwise low-quality plant or plant organ, thus aligning their nutritional intakes with their energetic requirements. Specifically, endophagous insects tend to avoid, or encounter lower amounts of, chemical and/or structural plant defenses that tend to concentrate in the cuticle and epidermis (Cornell, 1989). Many leaf-mining species, for example, consume nutrient-rich, internal mesophyll cells and do not eat epidermis and/or vascular tissues (Hering, 1951; Kimmerer and Potter, 1987; Body et al., 2015). Avoiding plant defenses and feeding on the most nutritious layers led to higher feeding efficiencies and higher performance of internal feeders compared to external feeders (Connor and Taverner, 1997; Giron et al., 2016).

Second, some endophagous larvae have also evolved specific morphological adaptations to cope with their confined nutritional niche and optimize their nutrition (Body et al., 2015). Hypermetamorphosis has been described in several lepidopteran leaf-miner species (e.g., Gracillariidae) and can be defined as a strong modification of larval morphology from one instar to the next associated with changes in feeding mode (Snodgrass, 1935). Evolution of this feeding strategy allows larvae to exploit over time different nutritional resources; therefore, early and late larval instars can occupy different feeding niches, providing superior nutrition by partitioning limited feeding resources within a confined nutritional space. Morphological adaptations, along with behavioral strategies, associated with hypermetamorphosis may also allow endophagous insects to avoid triggering plant defenses. Precise larval feeding may circumvent plant defenses that a clumsier feeding style might induce. For example, inconspicuous feeding targeting one or a few cell types (Djemai et al., 2001) may induce limited and/or transient plant defensive responses that have limited effects on herbivores, but this hypothesis still needs to be explicitly tested.

Beyond feeding styles, long-lasting interactions and intimate associations associated with endophagy are likely to have facilitated biochemical and hormonal crosstalk between internal-feeding insects and plants, setting the groundwork for host-plant manipulation by insects. Plant manipulation appears to provide an nutritional advantage because plant-manipulating insects are somehow able to concentrate nutrients and lower plant defenses in their food source, leading to higher insect performance and supporting the nutrition hypothesis for the adaptive nature of galls (Diamond et al., 2008; Stuart et al., 2012). Moreover, the manipulative ability of some endophagous insects may have facilitated various adaptive radiations of endophagy. By working from within plant tissue, some endophagous insects, particularly gall inducers and some leaf miners, are able to somehow ‘reprogram’ expression of the plant genome to force production of specialized nutritional resources that benefit the insect at the expense of plant growth and reproduction (Mothes and Engelbrecht, 1961; Giron et al., 2007; Diamond et al., 2008; Saltzmann et al., 2008; Kaiser et al., 2010; Giron et al., 2016). In fact, recent evidence suggests that gall-inducing species might be able to accomplish this reprogramming by synthesizing plant hormones, which alter host-plant physiology, including gene expression and host-plant defenses (Tooker and De Moraes, 2011a, b; Yamaguchi et al., 2012; Giron et al., 2016; Cambier et al., 2019). Conceivably, such manipulative traits may have played a role in adaptive radiations.

Notably, plant manipulation is not only restricted to gall inducers and leaf miners, as commonly assumed, but is shared by other endophagous insects (Stone and Schönrogge, 2003; Gutzwiller et al., 2015; Giron et al., 2016), and perhaps even ectophagous species (Andreas et al., 2020). Because endophagous insects secure their nutrition (and shelter) via their feeding habit, they also must evolve feeding strategies allowing them to meet their energetic and nutrient requirements, face variation in food and nutrient composition, and counteract plant defensive mechanisms. For example, larvae of European corn borer, *Ostrinia nubilalis*, can promote significant protein accumulation and elevated sugar and fatty-acid levels at their feeding site most likely due to effectors secreted by larvae (Dafoe et al., 2013). Contrary to gall-specific nutritive tissues where plant defenses are lowered (Stone and Schönrogge, 2003), stem borers appear to trigger plant-defense responses. However, increased levels of nutrients can override negative effects of plant chemical defenses (Dafoe et al., 2013) or larvae can potentially evolve effective tolerance or detoxification mechanisms against plant-produced defensive compounds. The intimate association between *O. nubilalis* and its host plant, including its nutritional limitations, may have selected for individuals that could alter nutritional resources while circumventing plant defenses.

These cases of endophytic species altering nutritional quality and/or defenses of host plants provide evidence that some insect species have evolved to exploit host-plant species for the nutrition that individuals need even if plants do not typical provide it, or enough of it. Should such an innovation arise, it is easy to imagine that selection would favor the trait, allowing it to spread across populations and perhaps lineages. Plant manipulation for nutritional purposes may thus have played a role in the evolution and diversification of endophagy.

### Competition

Competition is a key force that structures plant and animal communities; those individuals that gain competitive advantages for access to resources should succeed and reproduce. Despite some older ecological theory to the contrary (Hairston et al., 1960), competition is common among phytophagous insect species, including some endophytic species (Denno et al., 1995; Kaplan and Denno, 2007). However, little attention has focused on the potential role of competition for selecting for lineages to evolve internal feeding.

At first glance, one may not expect competition to influence internal feeders any differently than other sorts of herbivorous arthropods, but endophytic species, which are somewhat sessile, may be expected to compete even more strongly for their restricted resource than ectophytic species, which can often move to other food sources if they encounter competition (Denno et al., 1995). And a recent study found this to be the case; in particular, endophytic species appear to compete strongly with sap feeders (Bird et al., 2019). Therefore, once a lineage evolved an ability to be surrounded by plant tissue (e.g., boring, galling, mining, etc.), competition with some ectophytic species may have given endophytic species an advantage that may have first allowed the lineage to succeed and then to diversify. Indeed, competition among endophytic species appears to be quite common (Denno et al., 1995), suggesting that selection pressures may force endophytic species to partition resources to minimize competition (Bird et al., 2019). There is evidence of competition between free-living folivores and internal feeders (Denno et al., 1995; Kaplan and Denno, 2007; Bird et al., 2019), perhaps providing a glimpse of competitive interactions that may have encouraged endophagy, but such conclusions would be premature. In some of these interactions, the external feeder appears to have the competitive advantage, whereas in others the internal feeder does, making generalizations difficult (e.g., West, 1985; Fisher et al., 1999; Bird et al., 2019). We are unaware of any ecological evidence that suggests competition encouraged some taxa to adopt endophagy, which is an outcome over evolutionary time that seems plausible. Such scenarios may have to be inferred from phylogenies, but this would be challenging. Based on phylogenetic analyses, competition has been invoked as a factor that may have played a role in the shift from external feeding to internal feeding, including gall induction (Nyman et al., 2006) and in the transition from leaf rolling to gall induction (Guiguet, 2019), but the exact role of competition in these systems may be difficult to clarify.

While evidence for the role of competition in the evolution of endophagy may be scarce, some research supports competition as a force that could have increased the intimacy of interactions that some endophytic species have with their host-plant species. Some endophytic species (leaf-mining and stem-boring species) appear to have evolved an ability to manipulate their host plants to improve the local nutritional environment (Giron et al., 2007; Dafoe et al., 2013). Gall-inducing species, however, have evolved more intimate associations with their host plants and often can manipulate various aspects of plant morphology, chemistry, and physiology to improve their own success (Fay et al., 1993; Nyman and Julkunen-Tiitto, 2000; Stone and Schönrogge, 2003). Some of these manipulations appear to improve protection for gall inhabitants against invaders, whereas others decrease plant chemical defenses and/or improve nutritional quality (Nyman and Julkunen-Tiitto, 2000; Stone and Schönrogge, 2003; Tooker and De Moraes, 2007, 2009, 2011a, b; Tooker et al., 2008). Some evidence indicates that leaf miners and gall inducers can share the same host plant with other herbivores and avoid competitive exclusion by having different lifestyles. Indeed, even though gall-inducing and leaf-mining insects in early instars can both exploit the same resource, in later instars they can diverge to occupy different ecological niches within the same host plant (Guiguet, 2019), suggesting that niche partitioning to avoid competition may have been a strong evolutionary force leading to either form of endophagy.

Still other manipulations appear to give gall-inducing species advantages in competitive interactions with other herbivorous species. Often phenotypic changes associated with gall induction, such as altered plant physiology or chemistry, can extend beyond the gall to adjacent plant tissue, or may even extend to distant portions of the host plant, with effects that decrease the success of the other herbivorous species, but benefit the gall inducer (Schultz, 1992; Inbar et al., 1995; Foss and Rieske, 2004; Pascual-Alvarado et al., 2008; Prior and Hellmann, 2010; Rostás et al., 2013). For example, development of invasive gall wasp larvae on oaks negatively influenced foliar quality, which reduced performance of a native caterpillar species (Prior and Hellmann, 2010). Remarkably, gall-induced volatiles also can repel browsing mammals (Rostás et al., 2013). There are also examples of gall insects that have little influence on other herbivores on the same plant or even gall insects that facilitate more herbivory by other species (e.g., Fritz and Price, 1990; Nakamura et al., 2003), but the key to competitive advantage may relate to the manipulative capacity of the insect and associated sink strength.

The more resources that gall inducers tend to require from their host plants, the stronger the resource sink that they are likely to induce. Similarly, sink strength can potentially increase with more individuals feeding within a gall, or even more individuals infesting the same tissue. Competitive interactions between nutrient sinks have been largely overlooked. If demonstrated, this would be highly relevant for understanding the adaptive success of some endophytic strategies that can group tens of individuals on a single leaf (e.g., the horse-chestnut leafminer *Cameraria ohridella*) or in a single gall (e.g., gall-inducing social aphids or thrips). It may also shed light on evolution of sociality in endophytic insects as a way to optimize plant-nutrient interception against competition with plant and insect-induced sinks (Larson and Whitham, 1991, 1997). The strength of resource sinks appears to relate to the success of the gall inducer in competitive interactions with other herbivore species (Burstein et al., 1994; Inbar et al., 1995) or with plant sinks (Larson and Whitham, 1997). Further, it is logical then to expect that the stronger the resource needs of any gall-inducing species, the more likely it will have evolved manipulative tactics that give it an advantage in competitive interactions with other species. These tactics could involve altering host-plant chemistry, physiology, or morphology to negatively influence other herbivorous species. We propose that when the influence of gall insects reaches farther from the local vicinity of the gall that competition becomes increasingly relevant as a selective force that can shape the strength and direction of interactions with other herbivorous species. It is likely that revisiting nutrient allocation between various sinks through mass spectrometry imaging (Kaspar et al., 2011), tracing experiments, and manipulating sink strength with transplantation experiments and killing (Guiguet et al., 2018) will provide insight on the role of competition in the ecology and evolution of the endophagous lifestyle.

## Endophytic Taxa

Now that we have summarized some of the selection pressures that could have encouraged evolution of endophagy, we will consider the variety of endophagous feeding habitats that have evolved in six orders of herbivorous insects. For each taxon, we will then discuss which selection pressures that were likely to have played a role in the evolution and diversification of its endophytic groups. Because of similarities between selection pressures for Thysanoptera and Hemiptera, we discuss them together in one section, but treat the remaining taxa separately.

### Thysanoptera and Hemiptera

With their unique sucking mouthparts, thrips are not capable of burrowing into plant tissues to become endophytic (Figure 1 and Tables 1, 2). Nevertheless, endophagy has evolved multiple times within Thysanoptera, usually via tactics that allow thrips to attach leaves together or trigger plant responses that surround thrips in plant tissue (Mound and Morris, 2005). Ancestral families of thrips appear to be mycophagous, and this feeding habitat appears to be plesiomorphic (Grimaldi and Engel, 2005). Other groups of thrips feed upon flowers or leaves, and endophagy appears to have evolved, possibly multiple times, in each of these three lineages of thrips (Mound and Morris, 2005). Some endophytic thrips species feed within domiciles that they form by gluing together phyllodes, modified petioles that act as leaves, whereas other endophytic species are inquilines in galls induced by other thrips species (Morris et al., 1999).

Most endophytic thrips, however, are gregarious gall inducers, with many individuals contributing to gall induction (Ananthakrishnan, 1992). Lineages that include gall-inducing species also tend to include species whose feeding induces leaf crinkling, rolling, or folding, which are thought to be intermediate endophytic steps on the path to gall induction (Mound and Morris, 2005). Gall-inducing species tend to be monophagous on woody plant species in hot, dry portions of the Old World tropics, including Australia and Indo-Malaysia (Ananthakrishnan, 1992); thus, it seems likely that endophagy, and gall induction in particular, in many thrips species evolved as an adaptation to a persistent resource in challenging environments (Crespi et al., 1997; Grimaldi and Engel, 2005). Some gall-inducing taxa have also evolved advanced forms of sociality, including species that have soldier morphs (Crespi et al., 1997).

Ancestral hemipterans were plant feeders that ingested fluids (Grimaldi and Engel, 2005). Because of their characteristic sucking mouthparts, Hemiptera, similar to Thysanoptera, are unable to bore into plant tissue; thus, to feed endophytically various Hemiptera taxa have evolved feeding tactics that alter the structure of host-plant tissues and encase the feeding insect (Figure 1). Most endophytic Hemiptera tend to be gall-inducing species (Tables 1, 2). Galling is usually considered a derived feeding tactic, but within Hemiptera galling is also an ancient characteristic because it is represented in the most primitive group of psyllids (Burckhardt, 2005). Within some groups (e.g., psyllids and scale insects), the ability to induce galls appears to have evolved separately in multiple lineages (Burckhardt, 2005; Gullan et al., 2005).

Hemipteran gallers induce a diversity of galls, ranging from simple pit galls, to leaf-roll or fold galls to more complex covering galls, which may reflect degrees of evolutionary advancement (Figure 1; Yang and Mitter, 1994; Burckhardt, 2005; Gullan et al., 2005). Endophytic hemipterans can also be inquilines (Miller, 2005). Most species tend to be host-plant specific, often monophagous, but some gall-inducing scales insects are oligophagous or even polyphagous, though complex galls appear to be induced by species with more restricted host ranges (Gullan et al., 2005). In some cases oviposition can initiate galls, but typically nymph hemipterans induce galls and continued gall growth tends to require continuing nymphal feeding (Burckhardt, 2005). Endophytic feeding is uncommon in most hemipteran taxa. In aphids (Aphididae), for example, less than 10% of species are confirmed gall inducers (Wool, 2005).

As indicated above, Thysanoptera and Hemiptera have limited forms of endophagy. Most herbivorous species in these orders are external feeders and endophagy, has evolved a limited number of times, mostly as gall induction (Table 1). Of the three hypotheses that have been proposed to explain the adaptive significance of gall induction, the nutrition hypothesis has some of the strongest support for these two taxa, particularly for aphid and thrips galls (Stone and Schönrogge, 2003). For example, compared to ungalled leaves, aphid galls can provide increased concentrations of essential amino acids, which improve aphid performance (Koyama et al., 2004). Other hemipteran galls provide such a high quality diet that the gall-inducing inhabitants do not require bacterial endosymbionts, which help most hemipteran species process ingested food to satisfy dietary needs (Overholt et al., 2015). Similarly, in Australian thrips galls, galls with many internal folds have convergently evolved to provide superior nutrients to “hyperfecund” foundresses, suggesting that nutritional-based selection pressures have encouraged induction of resources that satisfy the nutrient needs of thrips females capable of producing high numbers of progeny (Crespi and Worobey, 1998).

Despite evidence supporting the importance of nutrition in evolution of gall induction for Thysanoptera and Hemiptera, other forces are likely to also have been at play. As mentioned above, the many thrips galls found in arid portions of Australia and Indo-Malaysia support the microenvironment hypothesis, suggesting that living inside plants may have decreased water stress that could prevent exterior feeders from thriving in hot dry environments (Ananthakrishnan, 1992; Crespi et al., 1997; Grimaldi and Engel, 2005). And for at least for some aphid species, competition may have contributed to evolution of the manipulative control that some galling aphids have over their host plant species. Galling aphids reap benefits of inducing strong nutrient sinks because they can extract the resources they need from their host plants while simultaneously depriving competing herbivores of resources they need (Burstein et al., 1994; Inbar et al., 1995).

### Hymenoptera

**FIGURE 2:**
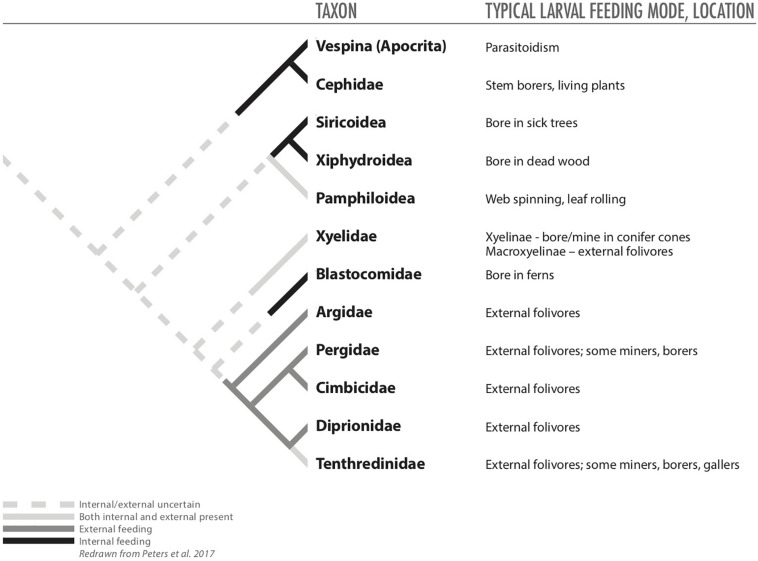
Phylogeny of basal Hymenoptera (from Peters et al., 2017) redrawn to illustrate hypothesized distributions of internal and external feeding among taxa.

Endophagy has been key to the evolution of groups within Hymenoptera, but it is unclear whether the earliest hymenopterans were endophagous or ectophagous herbivores (Figure 2; Sharkey, 2007; Peters et al., 2017). If they were ectophagous, among their first food sources could have been sporophylls of gymnosperms, as eaten by the extant family Xylidae (Grimaldi and Engel, 2005). Relatives of these early external feeders appear to have led to a radiation of ectophagous sawflies (i.e., Eusymphyta; Figures 1, 2), which includes the superfamilies Pamphilioidea and Tenthredinoidea (Peters et al., 2017). These groups include taxa that secondarily evolved a range of endophytic habits, including leaf miners, folders, and rollers and gall inducers (Table 2; Price, 1992; Connor and Taverner, 1997; Nyman et al., 1998). In particular, the family Tenthridinidae includes lineages that appear to have followed an evolutionary path from ectophagous leaf feeders to endophagous leaf folders and then gall inducers (Nyman et al., 1998, 2000). Within sawflies, the ability to induce a gall appears to have evolved independently six to ten times (Roininen et al., 2005). Some other early phytophagous hymenopterans (Xiphydriidae and Siricidae) also evolved endophagy as borers in dying or dead trees (Figure 2), and their lifestyle was facilitated by symbiotic fungi, which digest wood providing the wasp larvae more nutritious diets (Hanson, 1995; Solomon, 1995; Sharkey, 2007).

Despite some evidence that early hymenopterans were ectophagous, recent analyses raise the possibility that the common ancestor of symphytans and the remaining Hymenopterans (Eusymphyta + Unicalcarida) may have been an endophytic herbivore (Peters et al., 2017). Notably, once it evolved, the endophagous habit may have contributed to the diversification of the huge suborder of Apocrita (Figures 1, 2); endophytic taxa gave rise to carnivorous species that attacked other wood-boring Hymenoptera and Coleoptera, setting the path toward evolution of parasitoidism, which may have contributed to the success of Apocrita (Grimaldi and Engel, 2005; Sharkey, 2007).

Apocrita are largely carnivorous, but many apocritans have reverted to herbivory. None of these secondarily herbivorous taxa are external leaf feeders; they are all endophytic, feeding on nutritious plant tissues such as seeds, pollen, or gall tissue (including gallers and inquilines) or fungal tissue inside galls (Tables 1, 2; Hanson, 1995; La Salle, 2005; Wharton and Hanson, 2005). Some of these reversions to herbivory may have occurred via an intermediate step of entomophytophagous feeding, in which parasitoid species begin development by feeding upon their arthropod host and finish development by feeding upon plant tissue (La Salle, 2005). The next evolutionary step, of course, would be wasp species that feed only upon plant tissue. Other hymenopteran endophagous taxa appear to have evolved directly from phytophagous predecessors (Roskam, 1992; La Salle, 2005). Regardless of the path (Table 2), it is clear that endophagy, and more specifically gall induction, evolved many times in various apocritan taxa (Hanson, 1995; La Salle, 2005; Wharton and Hanson, 2005).

For Hymenoptera, ancestral species may have been endophytic, but this detail is unclear (Peters et al., 2017). Nevertheless, an endophytic lifestyle was established early in the evolution of Hymenoptera (Grimaldi and Engel, 2005). This internal feeding habit (i.e., boring in decaying wood) seems likely to have evolved because of nutritional benefits that could have been facilitated initially by symbiotic fungi, and later other types of symbiotic microbes, which provided access to previously inaccessible food resources (Hanson, 1995; Solomon, 1995; Sharkey, 2007). Moreover, the endophytic habit also appears to have been key for the evolution of parasitoidism, and for some endophytic Apocrita, like Agaonidae and Cynipidae (La Salle, 2005). Considering the closest non-herbivorous relatives of these taxa may provide insight on selective forces that led to their reversion to endophytic herbivory. Predecessors of both these endophagous groups (and others like Tanaostigmatidae) appear to be parasitoids (Peters et al., 2017); thus, prior to feeding upon plant tissue their relatives were already spending much of their lives inside hosts, which were embedded within plant tissues. As mentioned above, the switch to herbivory, therefore, could have been facilitated by entomophytophagous feeding, with fully herbivorous species evolving in a later step (La Salle, 2005). Because these groups are largely gall inducers, they likely later evolved their ability to manipulate their hosts to produce galls that provided even better nutritional resources (for agaonids, the enlarged endosperm of their galls; for cynipids, the nutritive tissues lining their galls; Weiblen, 2002; Csóka et al., 2005).

Of course other non-nutritional factors are likely to have been in play during evolution of endophagy in Hymenoptera. Some sawflies (e.g., Tenthredinoidea) appear to have followed a path in which ectophytic feeding appears to be ancestral and various forms of endophagy evolved later (Nyman et al., 2006). All the selection pressures involved in these transitions are not clear, but the natural-enemy hypothesis helps explain patterns of mortality documented within a gradation of external- to internal-feeding sawflies (Price and Pschorn-Walcher, 1988). Other studies with endophagous hymenopterans have found support for the microenvironment hypothesis (Miller et al., 2009), and benefits of endophagy for competition (Foss and Rieske, 2004).

### Coleoptera

Within Coleoptera, endophytic feeding achieves a diversity that exceeds that of Thysanoptera, Hemiptera, and Hymenoptera. Coleopterans can be borers, miners, gallers, and inquilines, and these endophytic species are most evident in the large superfamilies Chrysomeloidea and Curculionoidea (Tables 1, 2). This diversity of habits and abundance of species may be attributable in part to evolution of larvae with prognathous heads and chewing mouthparts, which would have allowed them to eat their way into plant tissue (Labandeira, 1997).

**FIGURE 3:**
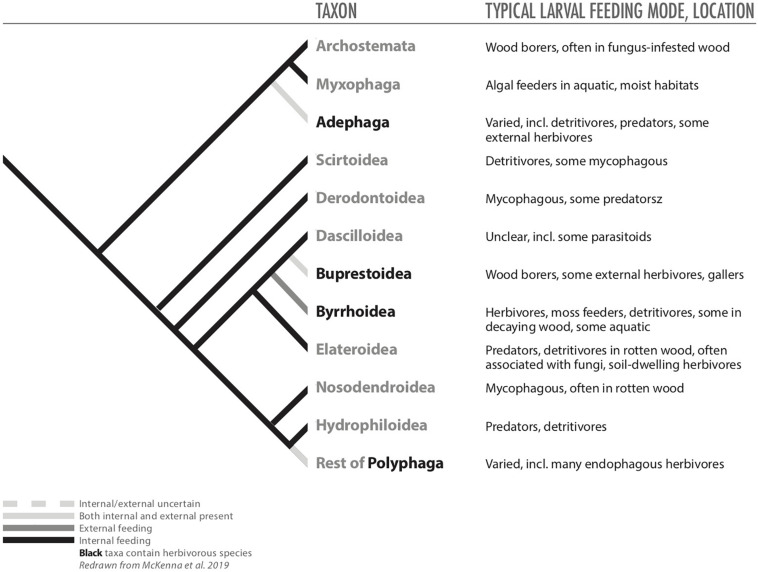
Phylogeny of basal Coleoptera (from McKenna et al., 2019) redrawn to illustrate hypothesized distributions of internal and external feeding among taxa.

In beetles, the endophytic habit appears to be derived from the ancestral state of boring in wood or other decaying tissues. Archostemata, one of the most basal suborders of Coleoptera, comprise families of specialized wood borers (Figures 1, 3; Grimaldi and Engel, 2005; McKenna et al., 2019). Larvae from early Permian beetles appear to have been associated with wood, similar to the extant families Ommatidae and Cupedidae, which are within Archostemata (Young, 2001; McKenna et al., 2019). The earliest fossil records of coleopteran wood boring are from the mid- to upper Permian (∼250 million years ago) in fungus-decayed wood, indicating that wood boring evolved early within Coleoptera (Feng et al., 2017; McKenna et al., 2019). Diversification of wood boring in beetles appears to have been facilitated by cellulolytic fungi that decomposed wood and ancestral beetle larvae appear to have fed upon the fungi, similar to modern ambrosia beetles, which independently converged on mycophagy (Massini et al., 2012; Feng et al., 2017; Hulcr and Stelinski, 2017). Saprophytic fungi, therefore, may have facilitated the transition of ancient beetles, or their predecessors, from feeding upon saprophytic fungi in leaf litter to borers feeding on similar fungi in decaying wood (Farrell, 1998; Grimaldi and Engel, 2005; Feng et al., 2017). Eventually endophytic beetle taxa evolved capacities to feed directly on wood and other tissues of living trees with its digestion facilitated by symbionts (Figure 3; Martin, 1991; Feng et al., 2017; Lieutier et al., 2017).

Recent evidence suggests that plant cell wall-degrading enzymes (PCWDE), which were acquired via horizontal gene transfer from bacteria and fungi that originated in detritus or insect guts, were a key innovation that facilitated success of beetles, particularly lineages whose larvae feed endophytically (McKenna et al., 2019). In Curculionoidea and Chrysomeloidea, for example, endophagy, apparently facilitated by PCWDE, may have been a key innovation that drove their diversification, allowing them to radiate inside a diversity of plant tissue and occupy novel niches (Farrell and Sequeira, 2004; Oberprieler et al., 2007; McKenna et al., 2019). Moreover, the abundance of endophagous species within these and other taxa may be explained in part by constraints imposed by morphological and behavioral traits associated with endophytic feeding; these traits may limit switches to other types of plant tissue, canalizing evolutionary trajectories (Farrell and Sequeira, 2004).

As larvae, the majority of endophytic Coleoptera taxa are associated with decaying, dying, or healthy plants, feeding within virtually all tissues (Table 1). Some taxa bore largely in herbaceous stems (e.g., Mordellidae; Jackman and Lu, 2002) or are specialized seed feeders (Chrysomelidae: Bruchinae; Kingsolver, 2002). Comparatively few major coleopteran taxa have evolved leaf-mining or gall-inducing habits (Table 1), which are often considered more derived endophytic feeding habits (Hering, 1951; Korotyaev et al., 2005). Among chrysomelids, however, seed boring by bruchine beetles appears to be the youngest or most derived endophagous habit, and seems to have evolved in a progression from stem feeding to gall inducing to seed boring (Farrell and Sequeira, 2004).

Because the diets of ancestral Coleoptera taxa may have been facilitated by fungi, microbial symbionts, or PCWDE (Martin, 1991; Farrell, 1998; Grimaldi and Engel, 2005; Feng et al., 2017; McKenna et al., 2019), the paths to endophagy may have been driven by nutritional selection pressures. The more derived taxa of Chrysomeloidea and Curculionoidea maintain this feeding habitat, which appear to be at least partly responsible for their success and diversification (Marvaldi et al., 2002). Beyond nutritional selection pressures, endophytic coleopteran populations must benefit from lower mortality from natural enemies associated with internal feeding (Hawkins et al., 1997) and likely gain advantages from being buffered from heat or moisture stress by being hidden within plant tissue, but we are unaware of explicit tests of these sorts of hypotheses with beetles.

### Lepidoptera

**FIGURE 4:**
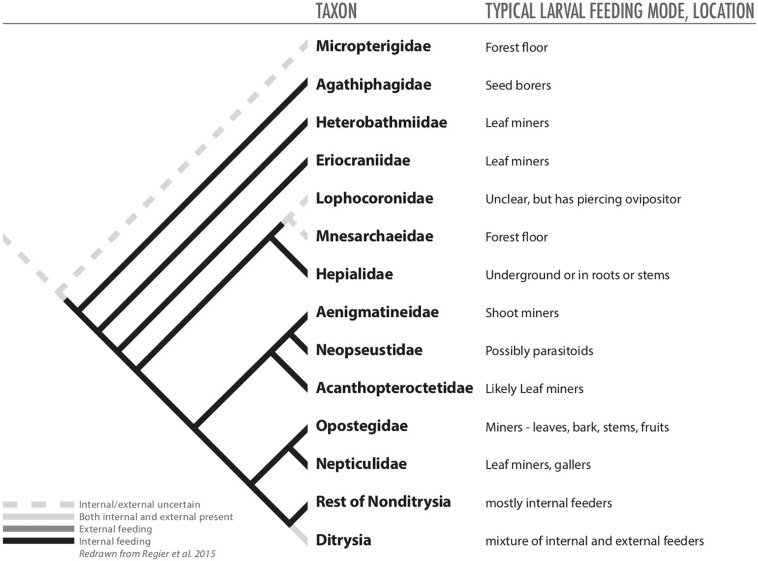
Phylogeny of basal Lepidoptera (from Regier et al., 2015) redrawn to illustrate hypothesized distributions of internal and external feeding among taxa.

Lepidoptera represents the largest diversification of herbivorous insects, and perhaps not surprisingly, also contains the highest diversity of endophagous habits, including many types of borers, concealed leaf feeders, leaf miners, gall inducers and inquilines (Table 2; Powell et al., 1998). Endophagy arose early in the evolution of Lepidoptera and may have fostered their subsequent radiation (Figure 4; Powell et al., 1998; Menken et al., 2010). Larvae of Micropterigidae appear to have fed on decaying tissue or live plants on the forest floor, but other basal lepidopterans adopted endophagy early in the evolution of Lepidoptera (Figures 1, 4; Regier et al., 2015). For example, larvae of Agathiphagidae are seed borers in pines of Araucariaceae, while larvae of Heterobathmiidae and Eriocraniidae mine leaves of tree species of Fagales (Kristensen et al., 2007; Regier et al., 2015).

Endophagy, therefore, was an early innovation in Lepidoptera that influenced the feeding habits of many non-ditrysian lineages (Regier et al., 2015). The endophagous habit further diversified onto angiosperms when they became available (Wiegmann et al., 2000; Menken et al., 2010) and specialized internal feeders begat larger insect taxa that fed as concealed external feeders (e.g., leaf rolling or similar), followed by radiations of fully exposed external feeders that achieved even larger sizes (Regier et al., 2013). In fact, the transition in Lepidoptera from endophagy to ectophagy may have been an “adaptive escape” from negative consequences of internal feeding, such as limits on body size, number of generations per year, access to alternative hosts, and leaf abscission (Powell et al., 1998). Notably, some extant taxa provide evidence of apparently “transitional” traits that combine endophytic and ectophytic habits. For example, some species of Adelidae and Incurvariidae feed internally in seeds and then switch to external feeding on fallen leaves (Powell et al., 1998). Species in the genus *Buccalatrix* (Bucculatricidae) move from leaf miners to external leaf feeders, while some gracilariids combine two different endophytic habits (*Caloptilia*, *Parornix*), feeding as miners for the first few instars and then become leaf folders (Hering, 1951; Nakadai and Kawakita, 2016). Other gracilariids first feed as leaf miners and then become gall inducers, and this transition involves hypermetamorphosis of mouthparts (Guiguet et al., 2018, 2019; Guiguet, 2019).

Because endophagy appears to have evolved early among lepidopterans, many taxa have had long associations with their host-plant taxa, allowing them to become specialized internal feeders. Indeed, some lepidopteran families, such as Nepticulidae, Gracillariidae, Cosmopterigidae, and Sesiidae, among others, are dominated by internal feeders (Powell et al., 1998). The long associations that many endophytic taxa have had with their host-plant species appears likely to contribute to most groups evolving some species capable of inducing galls (Table 1), which is considered a derived trait (Miller, 2005).

For Lepidoptera, the adoption of endophagy early in their evolution led to the large majority of non-ditrysian lineages taxa feeding inside plant tissue (Regier et al., 2015). Thus, nutrition, and perhaps exploiting empty feeding niches, may have been a primary factor in the success of early taxa. Moreover, recent evidence has demonstrated the high quality of endophagous tissue eaten by caterpillars, suggesting that internal feeding can give herbivores access to better sources of food (Diamond et al., 2008; Tooker and De Moraes, 2009; Giron et al., 2016). Nevertheless, these taxa likely gained other benefits from being inside plant tissues. Lepidopteran leaf miners appear to gain some protection from pathogens and predators by being hidden within plant tissue, but seem just as susceptible to parasitoid wasps as external feeders, perhaps discounting the value of the natural-enemies hypothesis for explaining the success of leaf mining within Lepidoptera (Connor and Taverner, 1997). We are not familiar with explicit tests of some of the other selection pressures that we have considered.

### Diptera

**FIGURE 5:**
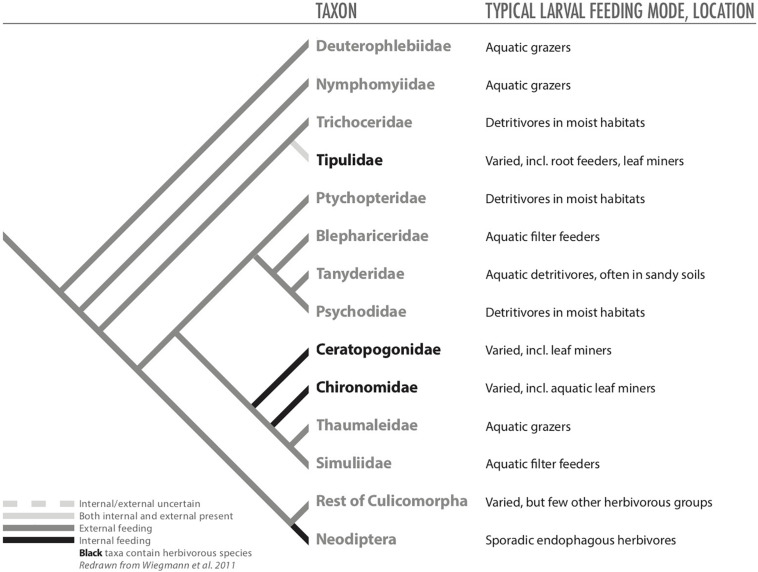
Phylogeny of basal Diptera (from Wiegmann et al., 2011) redrawn to illustrate hypothesized distributions of internal and external feeding among taxa.

Unlike its role in the evolution of the three other large groups of holometabolous insects (i.e., Hymenoptera, Coleoptera, and Lepidoptera), herbivory appears to have played a smaller role driving the basal patterns of evolution of Diptera (Grimaldi and Engel, 2005; Bertone et al., 2008; Wiegmann et al., 2011). The larvae of the most basal fly families are aquatic grazers, as it seems were ancestral dipterans with many species feeding upon algae (Figures 1, 5; Courtney, 1990; Wiegmann et al., 2011). Slightly more derived taxa are semi-aquatic and saprophagous (or even bacteriophagous) or mycophagous (Figure 5; Courtney, 1990; Dempewolf, 2005; Grimaldi and Engel, 2005; Wiegmann et al., 2011).

Nevertheless, herbivory in Diptera evolved at least 26 times, likely more than in any other order (Mitter et al., 1988), and is a dominant, derived feeding strategy within the order (Wiegmann et al., 2011). Remarkably, there are very few records of ectophagous herbivores within Diptera (possibly only in Tipulidae); therefore, plant feeding within flies appears to be synonymous with endophagy, with taxa that include borers (including seed feeders), miners, gallers, and inquilines (Labandeira, 2005; Table 1). These endophytic habits appear to have facilitated colonization of nutritional food sources, allowing fly larvae to remain in moist environments (i.e., avoid desiccation), eating liquid, or near liquid, diets, and allowing them to access nutrients despite having mouthparts poorly suited to chewing (Dempewolf, 2005). For some taxa, large radiations occurred across pteridophytes, gymnosperms, and angiosperms, resulting in ecologically and economically important groups, like Agromyzidae and Cecidomyiidae, which are dominated by mining and gall-inducing species, respectively, and are the most speciose taxa of endophytic Diptera (Labandeira, 2005). Beyond miners and gallers, Diptera contains relatively few borers, but they occur in all major clades of the order and appear to represent opportunistic exploitation of niches rather than an evolutionary radiation (Labandeira, 2005).

Dipteran miners are particularly notable for being a diverse guild that is well distributed taxonomically across the order from lower nemotocern dipterans (e.g., Culicomorpha: Chironomidae, Ceratopogonidae) to higher cyclorrhaphous flies (Muscoidea: Scathophagidae, Anthomyiidae; Labandeira, 2005). Mining appears to have evolved independently at least 25 times (Labandeira, 2005). Mining may even have been the initial entry into herbivory for Diptera (Dempewolf, 2005), but the fossil record appears unclear on this point because galling by flies is currently known from older deposits than mining by flies (Labandeira, 2005). Moreover, in some cases, mining appears to have been a predecessor to galling, but thus far there is limited evidence for this path to galling within Diptera (Dempewolf, 2005).

Diptera are also notable for containing one of the most unusual endophytic taxa, the Fergusoninidae. On their myrtaceous host plant species, these acalypterate flies have evolved complex, co-evolved lifecycles with mutualistic nematodes, which are deposited into meristematic tissue along with fly eggs and induce the galls in which maggots develop (Taylor et al., 2005). The maggot and nematodes feed together on plant tissue within the gall, then the worms enter female maggots where they become parasitic, eventually colonizing fly oviducts so they can be oviposited with the fly egg (Taylor et al., 2005). This mutualistic interaction is similar to relationships that other fly taxa (e.g., Cecidomyiidae) have with symbiotic fungi, which in some cases induce galls and then are feed upon by immature flies. In other cases, fungi occur in galls but only provide protection and do not appear to induce the gall or provide food (Gagné, 1989). Notably, plant feeding in cecidomyiids may have initially evolved from mycophagous ancestors (Roskam, 1992).

For Diptera, ancestral larval flies, and likely their progenitors, were aquatic grazers, and larvae of lower flies have remained faithful to aquatic or semi-aquatic habitats (Bertone et al., 2008). Even taxa that are largely saprophagous feed within liquid, or at least moist, habitats (e.g., decaying plant material in temporary pools, rotten wood; Bertone et al., 2008). Significantly, for each of the 26 times that herbivory has evolved within Diptera, the larval habitat has been endophagous; therefore, even among derived herbivorous fly taxa, species appear to be tied to moist environments inside plants. For hypothesizing which selection pressures played prominent roles in evolution of endophagy among Diptera, a parsimonious evolutionary explanation could be based on moist microenvironments (i.e., the microenvironment hypothesis), but a nutrition-based explanation could be just as likely because larval diets of flies are liquid, semi-liquid, or moist, as necessitated by the morphology of larval mouthparts (Labandeira, 2005). Importantly, once herbivory arose in dipteran taxa, how larvae fed upon plants and the nutrients they gained appears to have translated well to other tissues on the same plant or tissue of nearby plants, whether plant taxa were closely related or not, accounting in part for some of the species-level diversity in some fly taxa (Labandeira, 2005). Selection pressures associated with natural enemies seem less important because, as mentioned previously leaf-mining flies suffer high mortality from parasitoids wasps (Connor and Taverner, 1997), and gall flies do not necessarily gain more protection from larger galls (Waring and Price, 1989; Rossi et al., 1992; Abrahamson and Weis, 1997).

### Conclusion

After having considered endophagy in a much broader range of taxa than has been considered previously, we hypothesize that nutritional selection pressures played a primary role in the evolution of endophagy across orders of herbivorous insects. Given the general importance of nutritional resources to the success of animals, this hypothesis may not be surprising, but recurring support for it across orders is notable, as is the lack of consistent evidence supporting the other possible selection pressures. We must note, however, that nutritional hypotheses may just have received more attention in the literature rather than being more important for endophagy than the other factors we considered; further testing of the other explanatory hypotheses for the evolution of endophagy may reveal other patterns.

Because of its strong association with access to nutritional resources, competition imposed by the sedentary lifestyle of endophytic insects emerged from our analysis as a possible selective force in evolution of endophagy, and subsequent diversification and niche partitioning. This detail is noteworthy because we are unaware of previous consideration of competition as a selection pressure that encouraged endophagy in any form.

If nutritional selection pressures tend to be primary, then it seems reasonable to hypothesize that benefits associated with the other factors (e.g., microenvironment, attachment, natural enemies, and competition) would tend to be secondary, providing stronger or weaker advantages for certain insect taxa under some conditions. For example, under challenging environmental conditions it seems likely that endophagy is likely to provide benefits for water conservation. As mentioned above, galling tends to be more common in drier or hotter environments (Price et al., 1998), but similar analyses appear to be lacking for most other endophytic taxa. It would seem profitable, therefore, for future research to explore global patterns of endophagy to gain insight on the potential role of endophagy to limit heat and water stress. Testing these newly proposed hypotheses directly seems challenging, so it may be more feasible to test them indirectly in phylogenetic contexts, perhaps by characterizing water budgets in a range of taxa and feeding styles.

If a nutrition hypothesis best explains why so many insect taxa feed endophagously, it aligns well with the evidence available to explain the adaptive significance of more specialized forms of endophagy (Connor and Taverner, 1997; Stone and Schönrogge, 2003). As mentioned above, three hypotheses, nutrition, microenvironment, and natural enemies, have been proposed to explain the adaptive significance of leaf mining and insect galls. For leaf mining, it seems that the nutrition and microenvironment hypotheses best explain the advantages derived from mining (Connor and Taverner, 1997). However, as discussed above, analyses of Diptera revealed the dominance of endophagy across phytophagous groups, revealing that fly larvae are almost always associated with moist food sources, which aligns well with the capacity of their mouthparts (Dempewolf, 2005; Labandeira, 2005). These results appear to give more support to the nutritional hypothesis for helping to explain the adaptive significance of leaf mining, but we cannot overlook the potential interaction with microenvironment because fly larvae undoubtedly benefit from being surrounded by water filled tissue, and as a result may have been poorly adapted for external feeding. For insect gallers, the majority of the evidence also appears to support the role of nutrition and microenvironments for evolution of galling, and perhaps natural enemies have played a role in the morphological diversification of gall shapes and external features (Stone and Schönrogge, 2003). Given the nutritional support of endophagy provided by our review, we could also hypothesize that nutrition is the primary adaptive significance of galling and mining, and the other benefits are secondary, but further research will have to explore this sort of ranking.

As mentioned above, there is a tendency in ecological literature to believe that the progression of feeding habits in herbivorous insects started with external feeding and moved toward internal feeding. This belief is based on well-known theoretical and experimental work with sawflies (Price et al., 1987; Price and Pschorn-Walcher, 1988), but it may be the exception. Our review revealed that the evolutionary story is far more complicated, and varies by taxa (Table 2 and Figures 1–5). For some taxa, endophagy is an ancestral trait that has been around for hundreds of millions of years. For others, endophagy evolved more recently. The evidence we reviewed appears to indicate that nutritional benefits could underlie much of the evolution and diversification of endophagy across the orders of herbivorous insects. It is our hope that other researchers will now bring various research techniques to bear on these hypotheses to help clarify the evolutionary selection pressures involved in evolution of internal feeding.

## Author Contributions

Both authors listed have made a substantial, direct and intellectual contribution to the work, and approved it for publication.

## Conflict of Interest

The authors declare that the research was conducted in the absence of any commercial or financial relationships that could be construed as a potential conflict of interest.
